# Prognostic value of LECT2 and relevance to immune infiltration in hepatocellular carcinoma

**DOI:** 10.3389/fgene.2022.951077

**Published:** 2022-09-09

**Authors:** Jiangfeng Qin, Weijie Sun, Hui Zhang, Zihao Wu, Jiapei Shen, Wenhai Wang, Yuanyuan Wei, Yanyan Liu, Yufeng Gao, Honghai Xu

**Affiliations:** ^1^ Department of Pathology, The First Affiliated Hospital of Anhui Medical University, Hefei, China; ^2^ Department of Infectious Diseases, The First Affiliated Hospital of Anhui Medical University, Hefei, China; ^3^ Department of Pathology, the Fourth Affiliated Hospital of Anhui Medical University, Hefei, China; ^4^ Department of Hospital Infection Prevention and Control, the First Affiliated Hospital of Anhui Medical University, Hefei, China; ^5^ Anhui Center for Surveillance of Bacterial Resistance, Hefei, China

**Keywords:** leukocyte cell-derived chemotaxin2, HCC, immune, bioinformatics analysis, prognosis

## Abstract

**Background:** Previous studies have shown that Leukocyte cell-derived chemotaxin2 (LECT2) is associated with the development of HCC. However, there are still no studies with a comprehensive analysis of the role of LECT2 in hepatocellular carcinoma (HCC).

**Methods:** TCGA data sets were used to analyze the expression of LECT2 in HCC. In addition, the prognostic value of LECT2 in HCC was also investigated. DriverDBv3 was used to analyze the Mutation, CNV, and methylation profiles of LECT2. And, validated by immunohistochemistry in 72 HCC samples. The prognostic value of LECT2 and the correlation with clinicopathological features were analyzed. The GO/KEGG enrichment analysis of LECT2 co-expression and gene set enrichment analysis (GSEA) was performed using the R software package. The PPI interaction network was constructed by Search Tool for the Retrieval of Interacting Genes (STRING) database. Immune infiltration was estimated by the XCELL, TIMER, QUANTISEQ, MCPCOUNTER, EPIC, CIBERSORT abs and CIBERSORT algorithms, and Spearman was used to analyzing their correlation with LECT2. Moreover, we analyzed the correlation of LECT2 expression with immune checkpoint molecules and HLA genes. Finally, we analyzed the IC50 values of six chemotherapeutic drugs by the pRRophetic package.

**Results:** Reduced LECT2 expression levels found in HCC patients. Moreover, decreased levels of LECT2 were associated with poor overall survival, disease-free survival, disease-specific survival, and progression-free survival. Besides, methylation was significantly associated with LECT2 expression. The functional enrichment analysis revealed that LECT2 may affect HCC progression through various pathways such as JAK/STAT signaling pathway, cell cycle, and pathways in cancer. Additionally, the results showed that LECT2 expression was negatively correlated with immune infiltration of B cells, Neutrophil, Monocyte, Cancer-associated fibroblast, and Myeloid dendritic cell, and positively correlated with T cell CD8^+^ naive, Endothelial cell, and Hematopoietic stem cell. LECT2 expression was negatively correlated with multiple immune checkpoint molecules and HLA genes. Chemosensitivity analysis showed that chemosensitivity was lower in the LECT2 high expression group. We validated the prognostic value of LECT2 and analysis of clinicopathological features showed a lower TNM stage in the group with high expression of LECT2.

**Conclusion:** Low expression of LECT2 in HCC is closely associated with poor prognosis, LECT2 may have potential clinical applications due to its unique immunological effects.

## Introduction

Cancer is the leading cause of death in most countries of the world in the 21st century, and the increase in cancer incidence and mortality has caused widespread concern worldwide (Sung et al., 2021). Immunotherapy is one of the breakthroughs in cancer treatment and is becoming increasingly popular (Zhang and Zhang, 2020). However, a large number of cancer patients still have a poor prognosis due to distant metastases and cancer recurrence, with 5-years survival rates below 20% for many cancer subtypes (Bengtsson et al., 2020). Therefore, it is crucial to discover meaningful biomarkers to assess the prognosis of cancer.

Leukocyte cell-derived chemotaxin2 (LECT2) is a 16-kDa secreted protein (Ito et al., 2003), LECT2 was first reported as a chemotactic factor to promote the migration of neutrophils (Yamagoe et al., 1996). Recent evidence suggests that LECT2 is strongly associated with multiple disease progression, including renal amyloidosis (Comenzo, 2014), diabetes (Lan et al., 2014), and sepsis (Ando et al., 2012). In addition, identified as a hormone-like hepatokine, LECT2 is highly expressed in the liver (Zhu et al., 2022). Therefore, LECT2 is also closely associated with a variety of liver diseases, such as non-alcoholic fatty liver disease (NAFLD) (Yoo et al., 2017), and liver fibrogenesis (Xu et al., 2019), and hepatocellular carcinoma (Chen et al., 2016). We recently reported that LECT2 can suppress the migration and tube formations of endothelial cells via binding to Tie1 (Xu et al., 2019). Loss of LECT2 results in an increase of CD4^+^ T cells in the spleen (Greenow et al., 2018). By activating LPS signaling in macrophages, LECT2 links obesity to hepatic inflammation (Takata et al., 2021). All of this evidence suggests that LECT2 is closely associated with immune cell infiltration and may serve as a promising target for cancer immunotherapy.

Our study found that the expression level of LECT2 in HCC correlated with prognosis. According to the report, HCC progression is inhibited by LECT2 by controlling inflammatory monocytes (L'Hermitte et al., 2019). However, there is a lack of comprehensive studies on the prognostic value and the role of LECT2 in HCC in terms of immunotherapy. In this study, we investigated the expression and prognostic value of LECT2 in HCC. We also searched for possible signaling pathways by which LECT2 affects HCC and focused on exploring the correlation between LECT2 and immune infiltration. In addition to this, we performed a comprehensive analysis of the clinicopathological information of LECT2 in HCC patients. To our knowledge, this is the first study to analyze in detail the role of LECT2 in HCC, providing a reference for the use of LECT2 in HCC patients.

## Materials and methods

### Data download

The mRNA expression profile data from 33 different cancer patients from The Cancer Genome Atlas (TCGA, https://tcga-data.nci.nih.gov/tcga/), and missing data were removed information.

### Expression and survival analysis of LECT2

Using the limma package, we analyzed differential gene expression in 33 cancers and finally identified LECT2 with high expression levels in HCC and CHOL. Survival curves of high and low LECT2 expression level groups in HCC and CHOL were plotted using the Kaplan-Meier method to determine their prognostic value, with prognostic endpoints including overall survival (OS), disease-free survival (DFS), disease-specific survival (DSS) and progression-free survival (PFS).

### Exploring gene mutation, CNV (copy number variations), and methylation spectrum of LECT2

We explored the LECT2 gene mutation, CNV, and methylation spectrum using DriverDBv3 (http://driverdb.tms.cmu.edu.tw/). These data are developed and obtained for free.

### Enrichment analysis of LECT2 and PPI analysis

LinkedOmics database (http://www.linkedomics.org/login.php) is a fully functional multi-omics database that can be used for association analysis between genes (Vasaikar et al., 2018). LECT2 co-expression analysis was determined by Spearman correlation coefficients and displayed in the form of volcano and heat maps. Gene ontology (GO) function and Kyoto Encyclopedia of Genes and Genomes (KEGG) pathway enrichment analysis of co-expressed genes were performed by cluster profile package of R software, and we performed a visualization analysis of the data using the ggplot2 package. The PPI interaction network was constructed by Search Tool for the Retrieval of Interacting Genes (STRING) database (http://string-db.org/).

### Gene set enrichment analysis (GSEA)

GSEA is an approach that focuses on gene sets to explain biological pathways enriched by different populations (Subramanian et al., 2005). The samples were divided into high and low expression groups according to the median LECT2 expression, and then GSEA functional analysis was performed using the “limma”, “enrichplot”, “clusterProfiler” and “org.Hs.eg.db” packages. Gene set “c2. cp.kegg.v7.4. symbols.gmt” are obtained from GSEA website (https://www.gsea-msigdb.org/gsea/index.jsp).

### Immune infiltrate analysis

For a more comprehensive estimation of immune cell infiltration, we applied seven algorithms to estimate the immune cell infiltration status in the samples, including XCELL, TIMER, QUANTISEQ, MCPCOUNTER, EPIC, CIBERSORT abs, and CIBERSORT. These immune cell infiltration level results can be obtained from TIMER2.0 (Li et al., 2020) (http://timer.cistrome.org/). The relationship between LECT2 expression and immune infiltrating cells was calculated using Spearman correlation analysis. The significance threshold was set at *p* < 0.05.

### Correlation between LECT2 expression with immune checkpoint molecules and HLA genes

We calculated the relationship between LECT2 expression with 48 immune checkpoint molecules and 19 HLA genes using Spearman correlation analysis.

### Chemotherapy drug sensitivity analysis

We calculated the semi-inhibitory concentrations (IC50) of six commonly used chemotherapeutic drugs using the pRRophetic package to evaluate the sensitivity of HCC samples to the six chemotherapeutic drugs. IC50 difference between low and high expression groups was compared using Wilcoxon signed-rank test.

### Immunohistochemistry (IHC)

We examined 72 paraffin-embedded HCC tissues and adjacent tissue samples from the First Affiliated Hospital of Anhui Medical University using immunohistochemistry. These tissue specimens were obtained from patients who underwent liver resection from 2014 to 2015. All patients provided written informed consent and adhered to the Declaration of Helsinki. Ethical approval was obtained from the Ethics Committee of the First Affiliated Hospital of Anhui Medical University. Two experienced pathologists independently calculated immunohistochemical scores A score greater than or equal to three was considered high expression and less than three was considered low expression.

### Statistical analysis

In the comparison of clinicopathological features. Student’s t-test and Chi-square test were performed according to different types of variables. The results were considered statistically significant at a two-sided *p* < 0.05.

## Result

### LECT2 expression levels comparison

**FIGURE 1:**
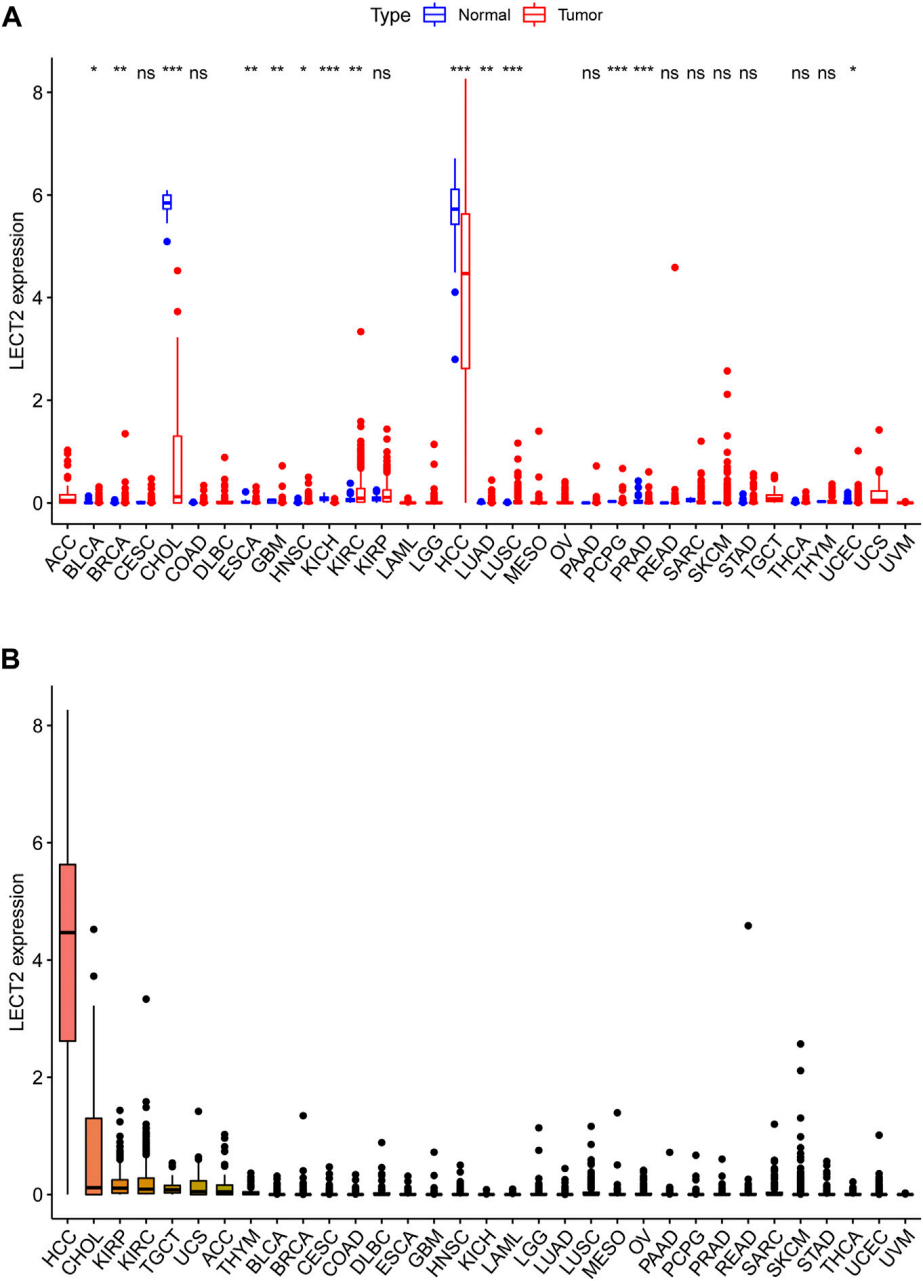
LECT2 expression levels comparison. **(A)** The differential expression analysis between tumor and normal groups of LECT2 in 33 cancers **(B)** The expression of LECT2 in 33 cancers (from high to low). “ns” represents no significance, "*" represents p < 0.05, "**" represents p < 0.01, and "***" represents p < 0.001 ".

The full names and abbreviations of the 33 cancers are shown in Supplementary Table S1. We analyzed the expression levels of LECT2 in 33 cancers in the TCGA database. Compared with the normal tissues, the results showed that LECT2 was differentially expressed in 14 of the 33 cancers (BLCA、BRCA、CHOL、ESCA、GBM、HNSC、KICH、KIRC、HCC、LUAD、LUSC、PCPG、PRAD、UCES). In particular, the expression levels of LECT2 were decreased in HCC and CHOL (Figure 1A). Meanwhile, we ranked the expression levels of LECT2 in 33 cancers and found that LECT2 had higher expression levels only in HCC and CHOL (Figure 1B).

### Prognostic value of LECT2

**FIGURE 2:**
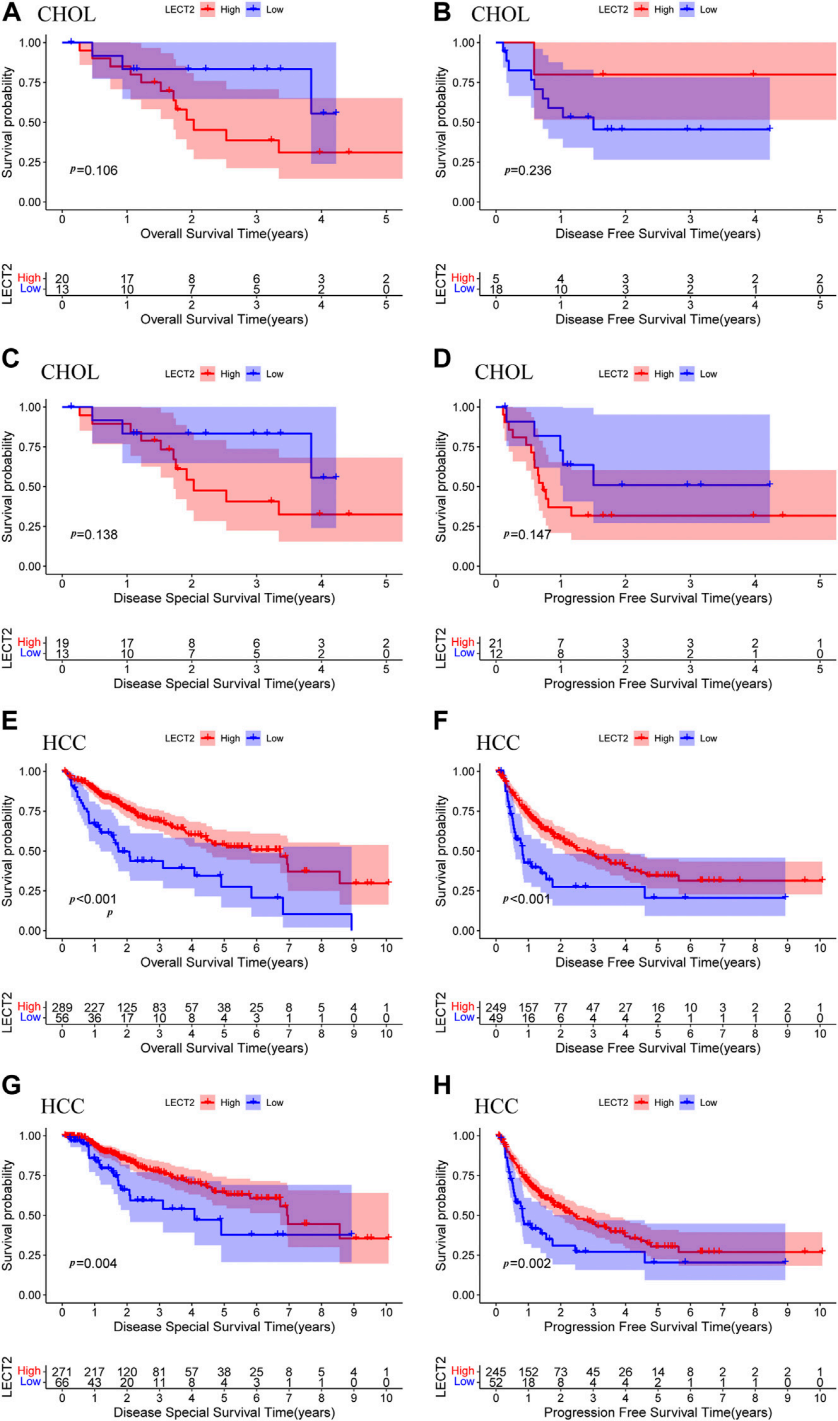
Prognostic analysis of LECT2. OS **(A)**, DFS **(B)**, DSS **(C)**, and PFS **(D)** of CHOL patients were grouped by high and low expression of LECT2. OS **(E)**, DFS **(F)**, DSS **(G)**, and PFS **(H)** of HCC patients were grouped by high and low expression of LECT2. OS: overall survival, DFS: disease-free survival, DSS: disease-specific survival, PFS: progression-free survival.

We further analyzed the prognostic value of LECT2 in cancers with differential expression. Because LECT2 is expressed only at high levels in CHOL and HCC and is too low in other cancers, we focused on the prognostic value of LECT2 in CHOL and HCC. Patients were divided into high expression group and low expression group, and we used Kaplan-Meier survival analysis to draw the survival curve of patients. In addition to OS, we also explored other important prognostic indicators such as DFS, DSS, and PFS. The results showed that the expression level of LECT2 did not affect the prognostic profile of CHOL patients, including OS (*p* = 0.106, Figure 2A), DFS (*p* = 0.236, Figure 2B), DSS (*p* = 0.138, Figure 2C) and PFS (*p* = 0.147, Figure 2D). However, the expression level of LECT2 significantly affected the prognosis of HCC patients. Patients with high expression of LECT2 had better OS (*p* < 0.001, Figure 2E), DFS (*p* < 0.001, Figure 2F), DSS (*p* = 0.004, Figure 2G) and PFS (*p* = 0.002, Figure 2H). The results showed that LECT2 is associated with the prognosis of HCC.

### Mutation, CNV, and methylation profiles of LECT2

**FIGURE 3:**
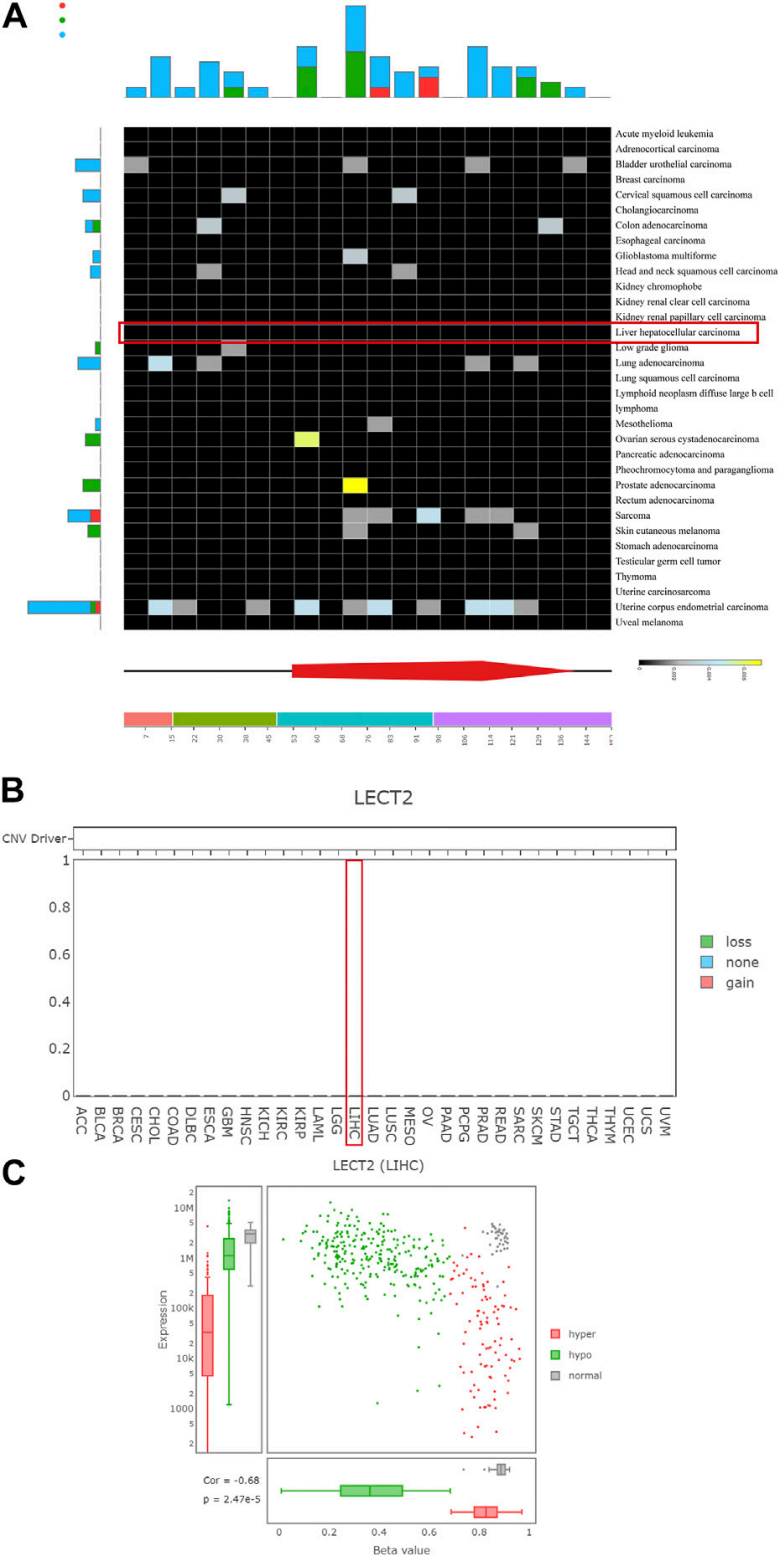
Mutation, CNV, and methylation profiles of LECT2. **(A)** The mutation status of LECT2 in various tumors according to the DriverDBv3 database **(B)** The CNV of LECT2 in various tumors **(C)** The methylation of LECT2 in HCC.

We further analyzed the possible reasons for the differential expression of LECT2 in HCC. To comprehensively analyze the mutational, CNV, and methylation spectrum of LECT2, we used DriverDBv3 to explore the mutational and CNV in all cancer types in the TCGA database. The DriverDBv3 database results showed no mutations and CNV of LECT2 in HCC (Figures 3A, B). Then we explored the correlation between LECT2 expression levels and methylation levels in HCC. The results showed that methylation levels were negatively correlated with LECT2 expression, with hypermethylation usually implying lower LECT2 expression (*p* < 0.001, Figure 3C). We think that methylation is one of the possible reasons for differential expression of LECT2 in HCC.

### Expression, prognostic value and clinicopathological features of LECT2 in HCC

**FIGURE 4:**
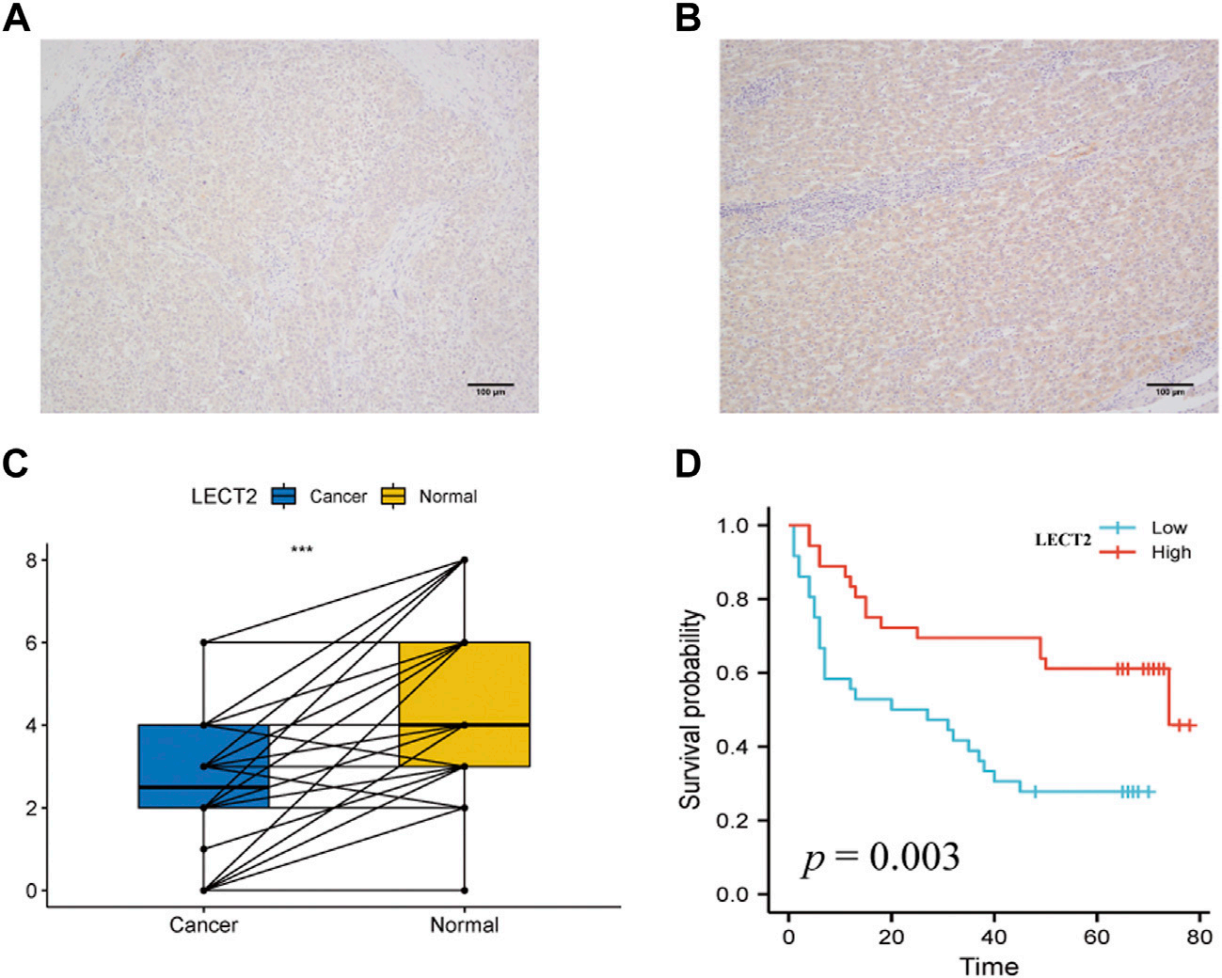
Expression levels and prognostic analysis of LECT2. **(A–B)** Representative IHC maps of LECT2 expression in HCC tissues and adjacent normal tissues **(C)** The expression levels of LECT2 in 72 cases of HCC and normal tissues, blue represents HCC tissue, yellow represents adjacent normal tissues **(D)** The prognostic analysis of LECT2 in 72 cases of HCC.

Given the unique expression pattern and prognostic value of LECT2 in HCC, we further validated the expression and prognostic value of LECT2 in HCC. representative IHC maps of LECT2 in HCC tissues and adjacent normal tissues are shown in Figures 4A,B. Further we examined the expression levels of LECT2 in 72 pairs of HCC and adjacent tissues. Consistent with the results of TCGA database, we found that the expression level of LECT2 was higher in adjacent tissues than in HCC tissues (Figure 4C). Meanwhile, we analyzed the prognostic and clinicopathological characteristics of 72 samples. The results showed that HCC patients in the high LECT2 expression group had better OS (*p* = 0.003, Figure 4D). In addition, the results showed that LECT2 expression levels correlated with TNM stage (*p* = 0.011), and TNM stage was higher in the low LECT2 expression group (Table 1).

**TABLE 1:** Clinicopathological features of LECT2 in HCC.

Factor	IHC score of LECT2		*p*-value
	High (n = 36)	Low (n = 36)	
Age, year	56.33 ± 11.50	57.67 ± 12.80	0.644
Sex			
Male	30 (83.3%)	30 (83.3%)	1.000
Female	6 (16.7%)	6 (16.7%)	
TNM stage			
I + II	33 (91.7%)	23 (63.9%)	**0.011**
III + IV	3 (8.3%)	13 (36.1%)	
Grade			
G1	8 (22.2%)	5 (13.9%)	0.634
G2	19 (52.8%)	22 (61.1%)	
G3	9 (25.0%)	9 (25.0%)	
The longest diameter of tumor	4.97 ± 3.35	5.89 ± 3.33	0.251

TNM, Tumor-Node-Metastasis. IHC, Immunohistochemistry.

### Enrichment analysis of LECT2 in HCC and PPI network analysis

**FIGURE 5:**
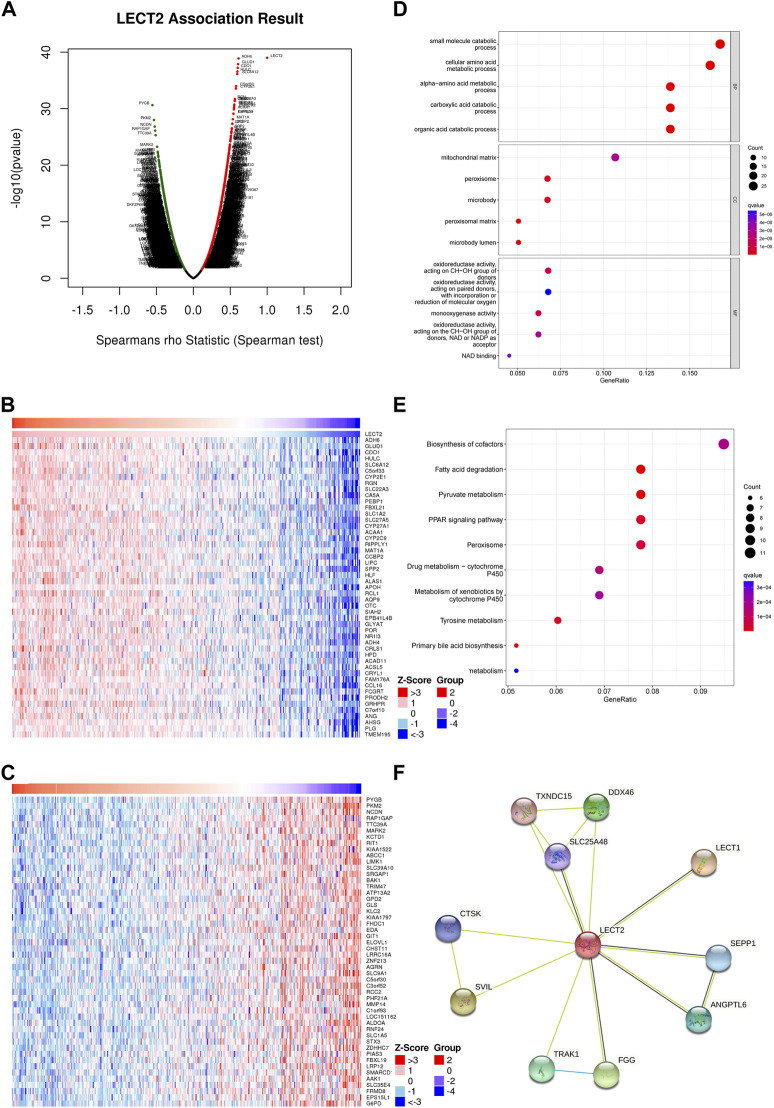
Enrichment analysis and PPI analysis of LECT2 in HCC. **(A)** Genes highly correlated with LECT2 identified in HCC by Spearman correlation analysis **(B)** Top 50 genes positively correlated with LECT2 in HCC. **(C)** Top 50 genes negatively correlated with LECT2 in HCC **(D)** Enrichment of gene ontology (GO) for genes correlated with LECT2. **(E)** Enrichment of Kyoto Encyclopedia of Genes and Genomes (KEGG) for genes correlated with LECT2 **(F)** Protein-protein interaction network of LECT2.

To find out the biological role of LECT2 in HCC, we analyzed the co-expression of LECT2 in HCC using the LinkedOmics database. As shown in Figure 5A, 3348 genes are positively related to LECT2, and 7,873 genes are negatively related to LECT2 (*p* < 0.05). The first 50 important genes that are positively (Figure 5B) and negatively (Figure 5C) correlated with LECT2 are shown in the heat map. After the GO and KEGG analysis of the top 200 co-expressed genes positively related to LECT2 expression. The top 15 results for GO including Biological Process (BP), Cellular Component (CC), and Molecular Function (MF) are shown in the bubble chart. Similarly, it showed the top 10 results for KEGG. The results of GO function showed that LECT2 co-expression is enriched in the small molecule catabolic process and cellular amino acid metabolic process (Figure 5D). The results of KEGG showed that LECT2 co-expression is correlated with Fatty acid degradation, Pyruvate metabolism, PPAR signaling pathway, and Peroxisome (Figure 5E). We studied the PPI network of LECT2 using the STRING database to learn more about the potential mechanisms of action of LECT2. We found that LECT2 is mainly related to LECT1 (0.775), SVIL (0.642), FGG (0.610), and DDX46 (0.601) which are the first four proteins (Figure 5F). The results showed that the expression of LECT2 is associated with several metabolic and disease pathways.

### GSEA of LECT2

**FIGURE 6:**
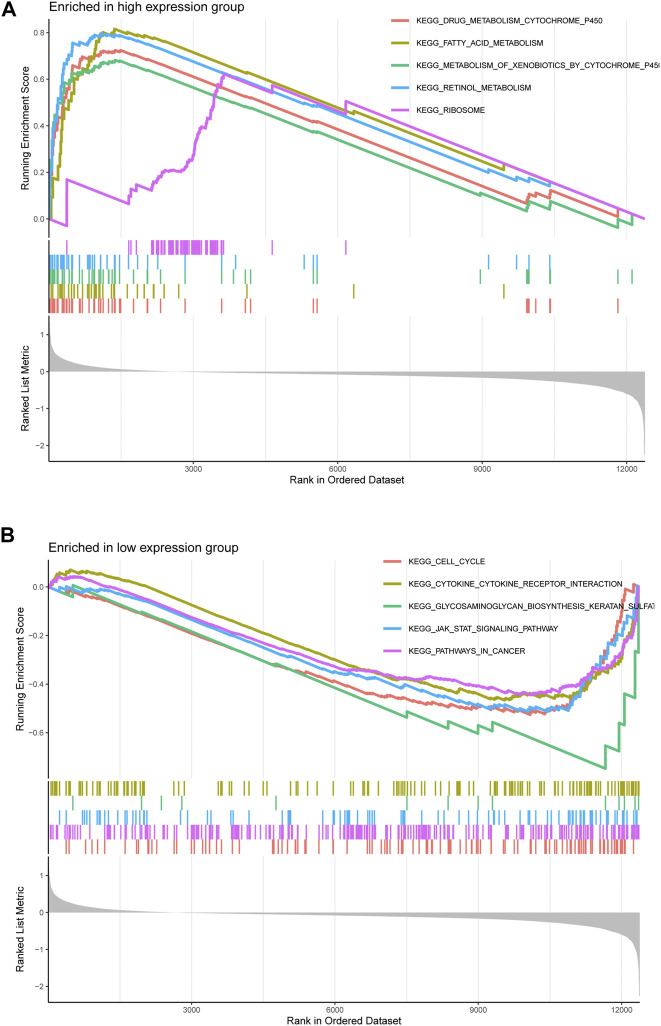
Gene Set Enrichment Analysis of LECT2 in HCC. **(A)** The five pathways were most significantly enriched in the LECT2 high-expression group **(B)** The five pathways were most significantly enriched in the LECT2 low-expression group.

Considering the strong correlation between LECT2 and HCC, we decided to investigate the potential pathways of LECT2 dysregulation in HCC.HCC patients were divided into high- and low-expression groups according to the median mRNA expression of LECT2 in the HCC cohort in TCGA. Further functional enrichment analyses showed in the LECT2 high expression group, the five most significant pathways, including KEGG DRUG METABOLISM CYTOCHROME P450, KEGG FATTY ACID METABOLISM, KEGG METABOLISM OF XENOBIOTICS BY CYTOCHROME P450, KEGG RETINOL METABOLISM and KEGG RIBOSOME were enriched (Figure 6A). In the LECT2 low expression group, the five most significant pathways, including KEGG CELL CYCLE, KEGG CYTOKINE RECEPTOR INTERACTION, KEGG GLYCOSAMINOGLYCAN BIOSYNTHESIS KERATAN SULFAT, KEGG JAK STAT SIGNALING PATHWAY and KEGG PATHWAYS IN CANCER were enriched (Figure 6B). Detailed GSEA analysis information is shown in Supplementary Table S2.

### Correlation between LECT2 and immune cells infiltrating

**FIGURE 7:**
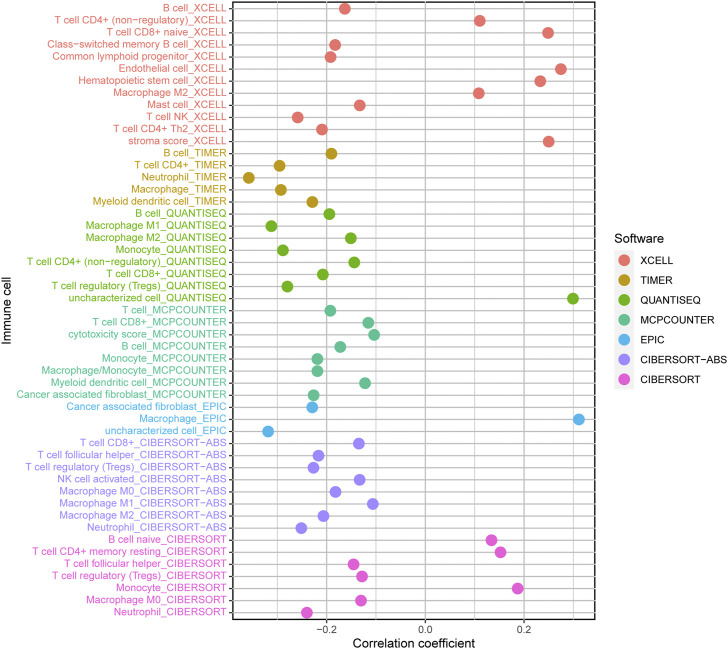
Correlations of LECT2 expression with immune infiltration level in HCC.

We further explored the relationship between LECT2 and the tumor immune microenvironment. The results showed that LECT2 expression was negatively correlated with immune infiltration of B cells, Neutrophil, Monocyte, Cancer-associated fibroblast, and Myeloid dendritic cell, and positively correlated with T cell CD8^+^ naive, Endothelial cell, and Hematopoietic stem cell. Meanwhile, the relationship between LECT2 expression and macrophages and macrophage M2 showed different results in different methods (Figure 7). The results suggested that Lect2 expression may affect the level of multiple immune cell infiltration in the tumor microenvironment of HCC.

### Correlation between LECT2 and predictive immune markers (Checkpoint and HLA) molecules

**FIGURE 8:**
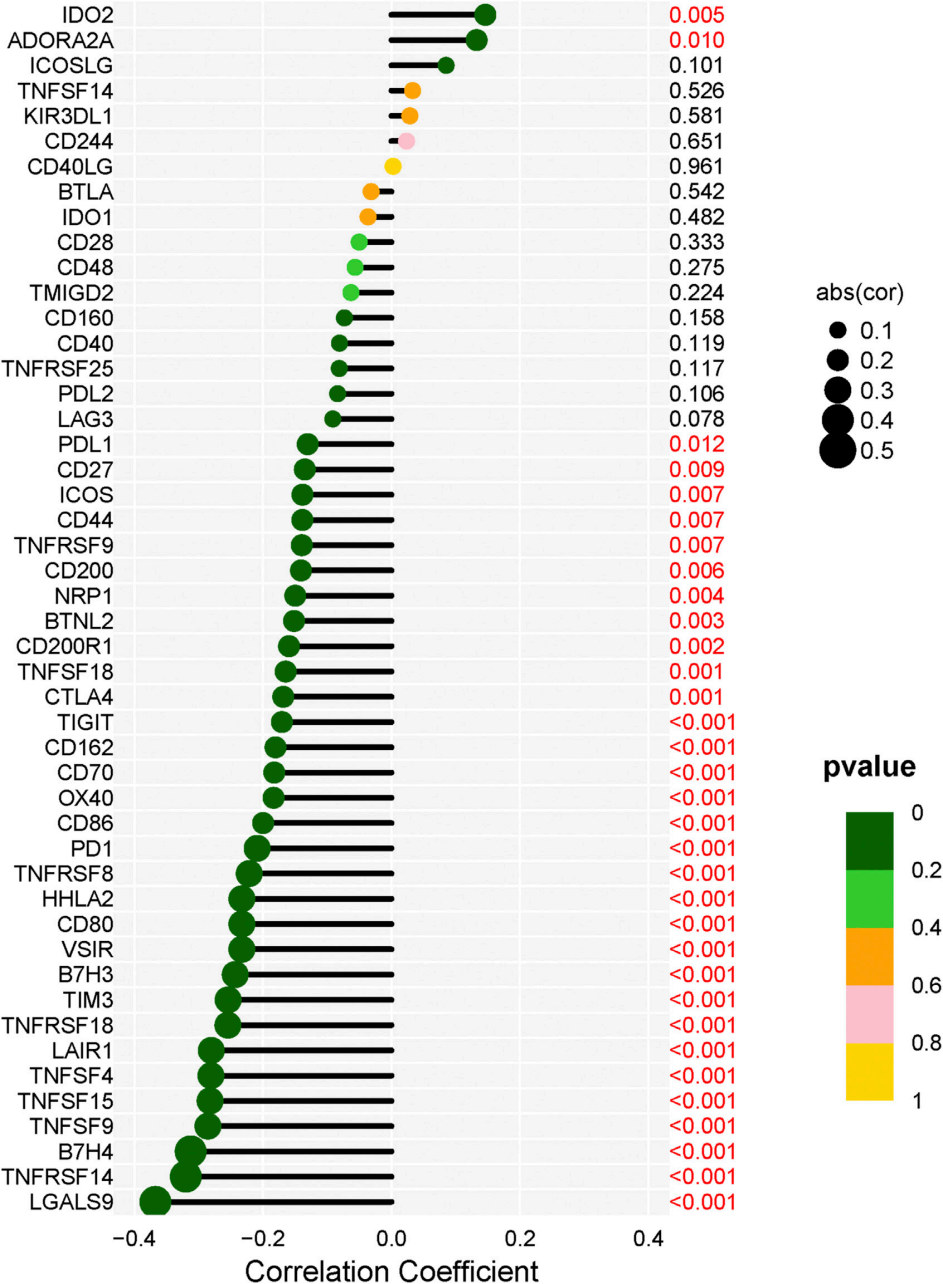
Correlation analysis of LECT2 expression with 48 immune checkpoint molecules.

**FIGURE 9:**
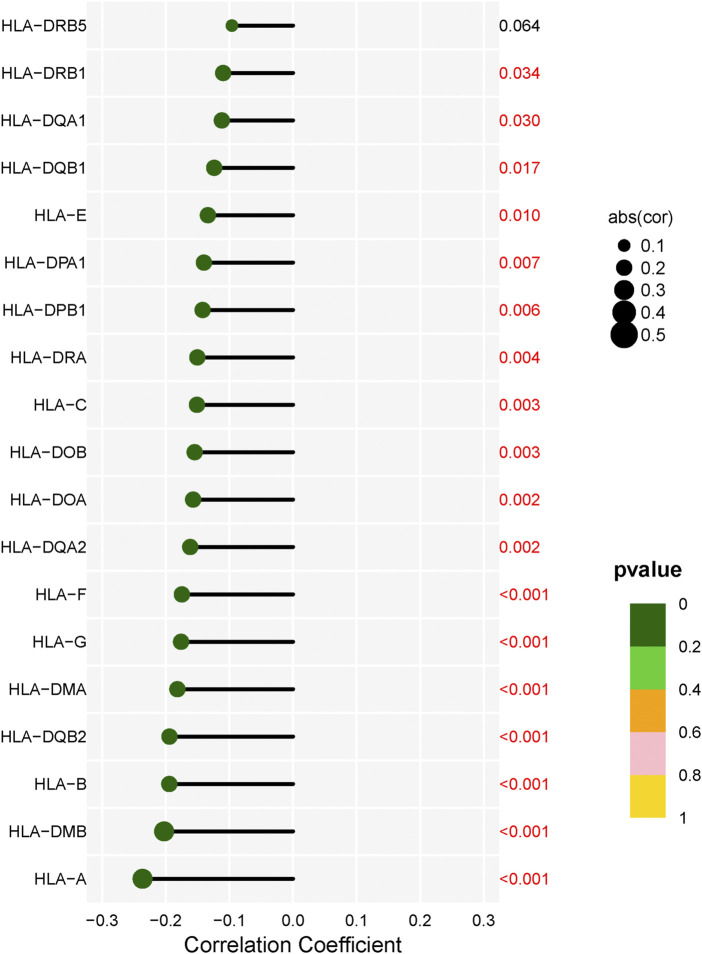
Correlation analysis of LECT2 expression with 19 HLA genes.

In addition, immunotherapy targeting immune checkpoint molecules is a promising target for immunotherapy in HCC patients. We then analyzed the relationship between LECT2 expression and 48 immune checkpoint molecules. We found that LECT2 was positively correlated with two immune checkpoint molecules, and negatively correlated with 31 immune checkpoint molecules (Figure 8). Therefore, HCC patients in the LECT2 low expression group may be more sensitive to immune checkpoint inhibitors, such as PD1 inhibitors and CTLA-4 inhibitors. HLA genes are important immune genes in the human body, and tumor-induced immune escape can change the expression of the HLA gene so that the tumor can evade the immune system without being killed (McGranahan et al., 2017). The results showed that LECT2 was negatively correlated with 18 of 19 HLA genes (Figure 9). In summary, LECT2 was negatively correlated with most of immune checkpoint molecules and HLA genes.

### Correlation analysis of LECT2 expression with chemotherapy drugs

**FIGURE 10:**
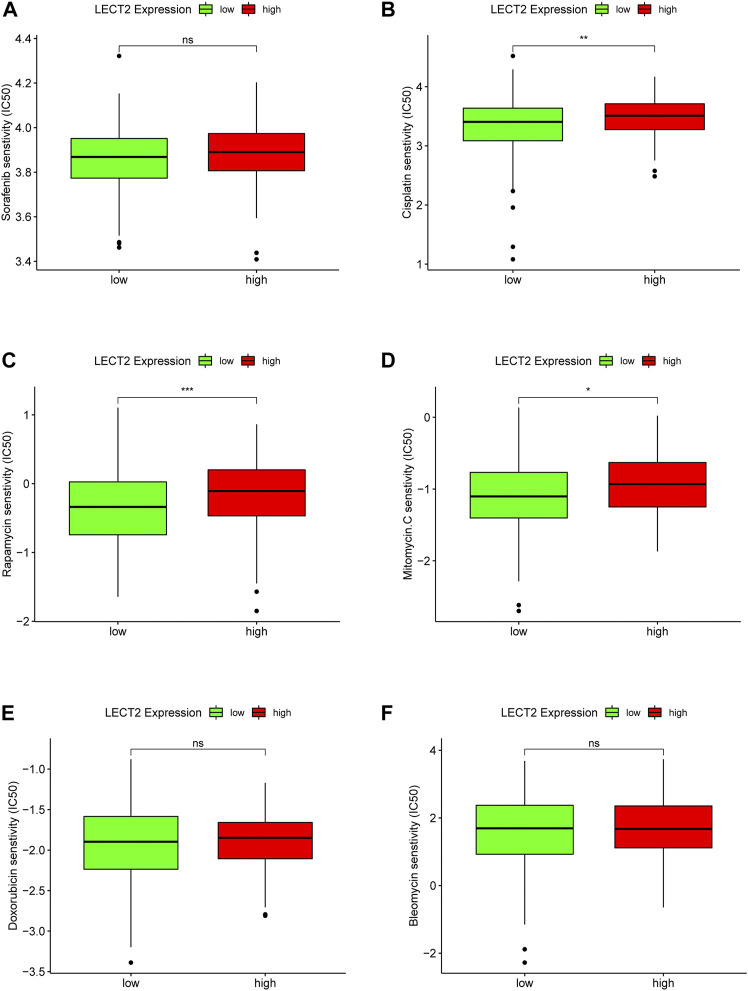
Correlation analysis of LECT2 expression with chemotherapeutic drug sensitivity. Difference analysis of the sensitivity of six chemotherapeutic drugs **(A)** Sorafenib, **(B)** Cisplatin, **(C)** Rapamycin, **(D)** Mitomycin, **(E)** Doxorubicin, **(F)** Bleomycin in LECT2 high expression group and low expression group, green represents low LECT2 expression, red represents high LECT2 expression. “ns” represents no significance, "*" represents p < 0.05, "**" represents p < 0.01, and "***" represents p < 0.001 ".

In addition, we also explored the relationship between the expression of LECT2 and the sensitivity of HCC patients to several common chemotherapeutic drugs. Sorafenib (Figure 10A), Cisplatin (Figure 10B), Rapamycin (Figure10C), Mitomycin (Figure 10D), Doxorubicin (Figure 10E), Bleomycin (Figure 10F). The results showed that high expression of LECT2 was associated with higher IC50 of Cisplatin (*p* < 0.01), Rapamycin (*p* < 0.001), and Mitomycin. C (*p* < 0.05) chemotherapy drugs (Figure 10). These results implied that patients with different LECT2 expression levels have different sensitivities to a variety of common chemotherapeutic drugs.

## Discussion

LECT2 is a 16-kDa secreted protein. It is mainly produced by hepatocytes (Yamagoe et al., 1998) and is usually expressed in vascular cells, endothelial cells, and VSMC (Slowik and Apte, 2017). A large number of studies have now shown that LECT2 is associated with the progression of a variety of cancers. For example, LECT2 is considered one of the potential prognostic risk biomarkers for colon adenocarcinoma (Yin et al., 2020). By inhibiting angiogenesis, LECT2 inhibits tumor growth in HCC (Chen et al., 2016). HCC with low LECT2 expression has a higher grade and inflammatory infiltrates (L'Hermitte et al., 2019). However, there are no systematic and comprehensive studies on the role of LECT2 in HCC. Therefore, there is a need to further explore the potential mechanisms of LECT2 in HCC. According to our knowledge, this is the first study to assess the role and significance of LECT2 in HCC regarding clinical, biological, and genomic aspects, laying the foundation for the clinical application of LECT2.

In the present study, we found higher expression levels of LECT2 in HCC and CHOL, and we reported significantly lower expression levels of LECT2 in HCC and CHOL samples compared to normal tissue. We further analyzed the prognostic value of LECT2 in HCC and CHOL. We found that LECT2 could affect both OS, DFS, DSS, and PFS in HCC patients and that low expression of LECT2 levels was a factor in poorer prognosis in HCC patients. Furthermore, we found that LECT2 mutations and CNV are uncommon times in HCC, but the abnormal expression of LECT2 may be due to abnormal methylation. Then, the results of 72 HCC clinical samples were consistent with the TCGA database, with HCC tissues having lower LECT2 levels than adjacent tissues. And the results showed that HCC patients in the LECT2 high expression group had better OS. Analysis of clinicopathological features showed a lower TNM stage in the group with high expression of LECT2. This suggests that high levels of LECT2 inhibit the progression of HCC.

Lu et al. found that LECT2 may inhibit HCC cell glycolysis during aerobic glycolysis, and reduced glycolysis by LECT2 might be linked to the inhibitory effect on HCC cells (Lu et al., 2020). However, there have been no studies on the functional enrichment analysis of LECT2 co-expression in HCC. In the present study, by GO and KEGG analysis of 200 genes associated with LECT2, we found that co-expression of LECT2 was mainly enriched in the small molecule catabolic process and cellular amino acid metabolic process. And we found that the LECT2 co-expression was mainly related to the Fatty acid degradation, Pyruvate metabolism, PPAR signaling pathway, and Peroxisome by KEGG analysis. It has been shown that the PPAR signaling pathway plays a key role in tumors (Wagner and Wagner, 2020). PPI analysis found that LECT2 has the strongest correlation with LECT1, SVIL, FGG, and DDX46. The study of LECT1 on osteosarcoma cells *in vivo* showed that it inhibited their growth and proliferation (Lin et al., 2017). Knockdown of DDX46 inhibited osteosarcoma cell proliferation and tumor growth *in vivo* (Jiang et al., 2017). SVIL (Houlier et al., 2020) and FGG (Peng et al., 2021) also have corresponding roles in the tumor process. At the same time, the GSEA pathway enrichment analysis showed that the JAK/STAT signaling pathway, cell cycle, and pathways in cancer were enriched in the low LECT2 expression group. Interestingly, blockade of the JAK/STAT signaling pathway mediated by SOCS3 was recently reported to inhibit the progression of HCC (Liu et al., 2021). These pathways may be potential mechanisms for LECT2 to regulate HCC. We suggest that LECT2 has other biological functions in HCC besides participating in glycolysis in HCC.

It is well documented that the tumor microenvironment plays an indispensable role in malignant tumors, and among them, immune cells are significant, and the level of various tumor immune cells affects the therapeutic effect (Hinshaw and Shevde, 2019). Therefore, the present study focused on exploring the correlation between tumor immune infiltration and LECT2. We used seven common methods for assessing immune cell infiltration. The results showed that LECT2 expression was negatively correlated with immune infiltration of B cells, Neutrophil, Monocyte, Cancer-associated fibroblast, and Myeloid dendritic cell, and positively correlated with T cell CD8^+^ naive, Endothelial cell, and Hematopoietic stem cell. The cancer-associated fibroblasts can increase angiogenesis, inflammation, proliferation, survival, EMT, and alter immune surveillance to promote HCC (Affo et al., 2017). Similarly, LECT2 loss contributes to the proliferation of inflammatory monocytes in HCC (L'Hermitte et al., 2019). These results are consistent with our analysis of the tumor suppressive role played by LECT2 in HCC, suggesting that LECT2 may regulate the progression of HCC by affecting these immune cells. In addition, our results showed that LECT2 was negatively correlated with 31 immune checkpoint molecules, including PD1 and CTLA-4, and was negatively correlated with 18 of 19 HLA genes. Moreover, in the LECT2 high expression group, the IC50 of chemotherapy drugs such as Cisplatin, Rapamycin, and Mitomycin. C was increased. In conclusion, these results provide a reference for the clinical use of drugs in HCC patients.

In conclusion, by comprehensively elucidating the expression, prognostic value, association with clinicopathological factors, co-expression network, pathway enrichment analysis, and crosstalk with immune infiltration in HCC, LECT2 may be a new potential prognostic and diagnostic biomarker for hepatocellular carcinoma with potential clinical applications.

## Conclusion

This study is the first to provide a comprehensive and detailed analysis of the role of LECT2 in HCC and to show that LECT2 is a new potential diagnostic and prognostic biomarker for hepatocellular carcinoma. However, further research is needed to explain the mechanisms of LECT2 involvement in HCC.

## Data Availability

The original contributions presented in the study are included in the article/supplementary material, further inquiries can be directed to the corresponding authors.

## References

[B1] Affo S. Yu L. X. Schwabe R. F. (2017). The role of cancer-associated fibroblasts and fibrosis in liver cancer. Annu. Rev. Pathol. 12, 153–186. 10.1146/annurev-pathol-052016-100322 27959632 PMC5720358

[B2] Ando K. Kato H. Kotani T. Ozaki M. Arimura Y. Yagi J. (2012). Plasma leukocyte cell-derived chemotaxin 2 is associated with the severity of systemic inflammation in patients with sepsis. Microbiol. Immunol. 56 (10), 708–718. 10.1111/j.1348-0421.2012.00488.x 22725643

[B3] Bengtsson A. Andersson R. Ansari D. (2020). The actual 5-year survivors of pancreatic ductal adenocarcinoma based on real-world data. Sci. Rep. 10 (1), 16425. 10.1038/s41598-020-73525-y 33009477 PMC7532215

[B4] Chen C. K. Yu W. H. Cheng T. Y. Chen M. W. Su C. Y. Yang Y. C. (2016). Inhibition of VEGF165/VEGFR2-dependent signaling by LECT2 suppresses hepatocellular carcinoma angiogenesis. Sci. Rep. 6, 31398. 10.1038/srep31398 27507763 PMC4979047

[B5] Comenzo R. L. (2014). LECT2 makes the amyloid list. Blood 123 (10), 1436–1437. 10.1182/blood-2014-01-549758 24627547

[B6] Greenow K. R. Zverev M. May S. Kendrick H. Williams G. T. Phesse T. (2018). Lect2 deficiency is characterised by altered cytokine levels and promotion of intestinal tumourigenesis. Oncotarget 9 (92), 36430–36443. 10.18632/oncotarget.26335 30559928 PMC6284865

[B7] Hinshaw D. C. Shevde L. A. (2019). The tumor microenvironment innately modulates cancer progression. Cancer Res. 79 (18), 4557–4566. 10.1158/0008-5472.CAN-18-3962 31350295 PMC6744958

[B8] Houlier A. Pissaloux D. Masse I. Tirode F. Karanian M. Pincus L. B. (2020). Melanocytic tumors with MAP3K8 fusions: Report of 33 cases with morphological-genetic correlations. Mod. Pathol. 33 (5), 846–857. 10.1038/s41379-019-0384-8 31719662

[B9] Ito M. Nagata K. Kato Y. Oda Y. Yamagoe S. Suzuki K. (2003). Expression, oxidative refolding, and characterization of six-histidine-tagged recombinant human LECT2, a 16-kDa chemotactic protein with three disulfide bonds. Protein Expr. Purif. 27 (2), 272–278. 10.1016/s1046-5928(02)00634-4 12597887

[B10] Jiang F. Zhang D. Li G. Wang X. (2017). Knockdown of DDX46 inhibits the invasion and tumorigenesis in osteosarcoma cells. Oncol. Res. 25 (3), 417–425. 10.3727/096504016X14747253292210 27697093 PMC7841134

[B11] L'Hermitte A. Pham S. Cadoux M. Couchy G. Caruso S. Anson M. (2019). Lect2 controls inflammatory monocytes to constrain the growth and progression of hepatocellular carcinoma. Hepatology 69 (1), 160–178. 10.1002/hep.30140 30070727

[B12] Lan F. Misu H. Chikamoto K. Takayama H. Kikuchi A. Mohri K. (2014). LECT2 functions as a hepatokine that links obesity to skeletal muscle insulin resistance. Diabetes 63 (5), 1649–1664. 10.2337/db13-0728 24478397

[B13] Li T. Fu J. Zeng Z. Cohen D. Li J. Chen Q. (2020). TIMER2.0 for analysis of tumor-infiltrating immune cells. Nucleic Acids Res. 48 (W1), W509–W514. 10.1093/nar/gkaa407 32442275 PMC7319575

[B14] Lin X. Wang L. Wang F. (2017). ChondromodulinI suppresses tumorigenesis of human osteosarcoma cells. Mol. Med. Rep. 16 (6), 8542–8548. 10.3892/mmr.2017.7629 28983591

[B15] Liu Z. K. Li C. Zhang R. Y. Wei D. Shang Y. K. Yong Y. L. (2021). EYA2 suppresses the progression of hepatocellular carcinoma via SOCS3-mediated blockade of JAK/STAT signaling. Mol. Cancer 20 (1), 79. 10.1186/s12943-021-01377-9 34044846 PMC8157759

[B16] Lu C. Fang S. Weng Q. Lv X. Meng M. Zhu J. (2020). Integrated analysis reveals critical glycolytic regulators in hepatocellular carcinoma. Cell. Commun. Signal. 18 (1), 97. 10.1186/s12964-020-00539-4 32576292 PMC7310503

[B17] McGranahan N. Rosenthal R. Hiley C. T. Rowan A. J. Watkins T. B. K. Wilson G. A. (2017). Allele-specific HLA loss and immune escape in Lung cancer evolution. Cell. 171 (6), 1259–1271. e1211. 10.1016/j.cell.2017.10.001 29107330 PMC5720478

[B18] Peng H. H. Wang J. N. Xiao L. F. Yan M. Chen S. P. Wang L. (2021). Elevated serum FGG levels prognosticate and promote the disease progression in prostate cancer. Front. Genet. 12, 651647. 10.3389/fgene.2021.651647 33995485 PMC8117098

[B19] Slowik V. Apte U. (2017). Leukocyte cell-derived chemotaxin-2: It's role in pathophysiology and future in clinical medicine. Clin. Transl. Sci. 10 (4), 249–259. 10.1111/cts.12469 28466965 PMC5504477

[B20] Subramanian A. Tamayo P. Mootha V. K. Mukherjee S. Ebert B. L. Gillette M. A. (2005). Gene set enrichment analysis: A knowledge-based approach for interpreting genome-wide expression profiles. Proc. Natl. Acad. Sci. U. S. A. 102 (43), 15545–15550. 10.1073/pnas.0506580102 16199517 PMC1239896

[B21] Sung H. Ferlay J. Siegel R. L. Laversanne M. Soerjomataram I. Jemal A. (2021). Global cancer statistics 2020: GLOBOCAN estimates of incidence and mortality worldwide for 36 cancers in 185 countries. Ca. Cancer J. Clin. 71 (3), 209–249. 10.3322/caac.21660 33538338

[B22] Takata N. Ishii K. A. Takayama H. Nagashimada M. Kamoshita K. Tanaka T. (2021). LECT2 as a hepatokine links liver steatosis to inflammation via activating tissue macrophages in NASH. Sci. Rep. 11 (1), 555. 10.1038/s41598-020-80689-0 33436955 PMC7804418

[B23] Vasaikar S. V. Straub P. Wang J. Zhang B. (2018). LinkedOmics: Analyzing multi-omics data within and across 32 cancer types. Nucleic Acids Res. 46 (D1), D956–D963. 10.1093/nar/gkx1090 29136207 PMC5753188

[B24] Wagner N. Wagner K. D. (2020). PPAR beta/delta and the hallmarks of cancer. Cells 9 (5), E1133. 10.3390/cells9051133 32375405 PMC7291220

[B25] Xu M. Xu H. H. Lin Y. Sun X. Wang L. J. Fang Z. P. (2019). LECT2, a ligand for Tie1, plays a crucial role in liver fibrogenesis. Cell. 178 (6), 1478–1492. 10.1016/j.cell.2019.07.021 31474362

[B26] Yamagoe S. Mizuno S. Suzuki K. (1998). Molecular cloning of human and bovine LECT2 having a neutrophil chemotactic activity and its specific expression in the liver. Biochim. Biophys. Acta 1396 (1), 105–113. 10.1016/s0167-4781(97)00181-4 9524238

[B27] Yamagoe S. Yamakawa Y. Matsuo Y. Minowada J. Mizuno S. Suzuki K. (1996). Purification and primary amino acid sequence of a novel neutrophil chemotactic factor LECT2. Immunol. Lett. 52 (1), 9–13. 10.1016/0165-2478(96)02572-2 8877413

[B28] Yin Z. Yan X. Wang Q. Deng Z. Tang K. Cao Z. (2020). Detecting prognosis risk biomarkers for colon cancer through multi-omics-based prognostic analysis and target regulation simulation modeling. Front. Genet. 11, 524. 10.3389/fgene.2020.00524 32528533 PMC7264416

[B29] Yoo H. J. Hwang S. Y. Choi J. H. Lee H. J. Chung H. S. Seo J. A. (2017). Association of leukocyte cell-derived chemotaxin 2 (LECT2) with NAFLD, metabolic syndrome, and atherosclerosis. PLoS One 12 (4), e0174717. 10.1371/journal.pone.0174717 28376109 PMC5380318

[B30] Zhang Y. Zhang Z. (2020). The history and advances in cancer immunotherapy: Understanding the characteristics of tumor-infiltrating immune cells and their therapeutic implications. Cell. Mol. Immunol. 17 (8), 807–821. 10.1038/s41423-020-0488-6 32612154 PMC7395159

[B31] Zhu S. Bennett S. Li Y. Liu M. Xu J. (2022). The molecular structure and role of LECT2 or CHM-II in arthritis, cancer, and other diseases. J. Cell. Physiol. 237 (1), 480–488. 10.1002/jcp.30593 34550600

