# Supplementary material for: Prognostic value of LECT2 and relevance to immune infiltration in hepatocellular carcinoma
Source: Front Genet. 2022 Sep 9;13:951077. doi: 10.3389/fgene.2022.951077 (PMC9500357; doi:10.3389/fgene.2022.951077)
Supplement: Supplementary file 1 [file Table1.DOCX]

**Supplemental table S1. 33 types of human cancers employed in our research.**

| **Abbreviation** | **Full name** |
| --- | --- |
| ACC  BLCA | Adrenocortical carcinoma |
|  | Bladder urothelial carcinoma |
| BRCA  CESC | BRCA Breast invasive carcinoma |
|  | Cervical squamous cell carcinoma and endocervical |
| CHOL | adenocarcinoma |
|  | Cholangiocarcinoma |
| COAD  DLBC | Colon adenocarcinoma |
|  | Lymphoid neoplasm diffuse large B-cell lymphoma |
| ESCA  GBM | Esophageal carcinoma |
|  | Glioblastoma multiforme |
| HNSC  KICH | Head and neck squamous cell carcinoma |
|  | Kidney chromophobe |
| KIRC  KIRP | Kidney renal clear cell carcinoma |
|  | Kidney renal papillary cell carcinoma |
| LAML  LGG | Acute myeloid leukemia |
|  | Brain lower grade glioma |
| LIHC  LUAD | Liver hepatocellular carcinoma |
|  | Lung adenocarcinoma |
| LUSC  MESO | Lung squamous cell carcinoma |
|  | Mesothelioma |
| OV | Ovarian serous cystadenocarcinoma |
| PAAD | Pancreatic adenocarcinoma |
| PCPG | Pheochromocytoma and paraganglioma |
| PRAD | Prostate adenocarcinoma |
| READ | Rectum adenocarcinoma |
| SARC | Sarcoma |
| SKCM | Skin cutaneous melanoma |
| STAD | Stomach adenocarcinoma |
| TGCT | Testicular germ cell tumors |
| THCA | Thyroid carcinoma |
| THYM | Thymoma |
| UCEC | Uterine corpus endometrial carcinoma |
| UCS | Uterine carcinosarcoma |
| UVM | Uveal melanoma |
